# Supplementary material for: Loss and Gain of Gut Bacterial Phylotype Symbionts in Afrotropical Stingless Bee Species (Apidae: Meliponinae)
Source: Microorganisms. 2021 Nov 24;9(12):2420. doi: 10.3390/microorganisms9122420 (PMC8708602; doi:10.3390/microorganisms9122420)
Supplement: Supplementary file 1 [file microorganisms-09-02420-s001.zip › Y. H. Tola SUP figures.pdf]

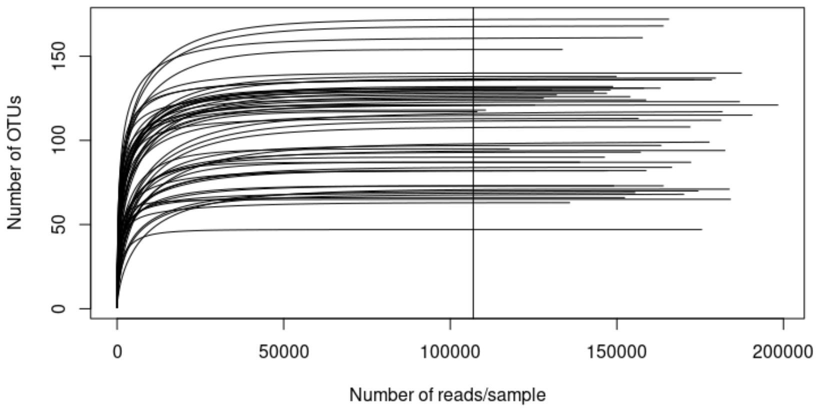

Figure S1: Alpha rarefaction curve.

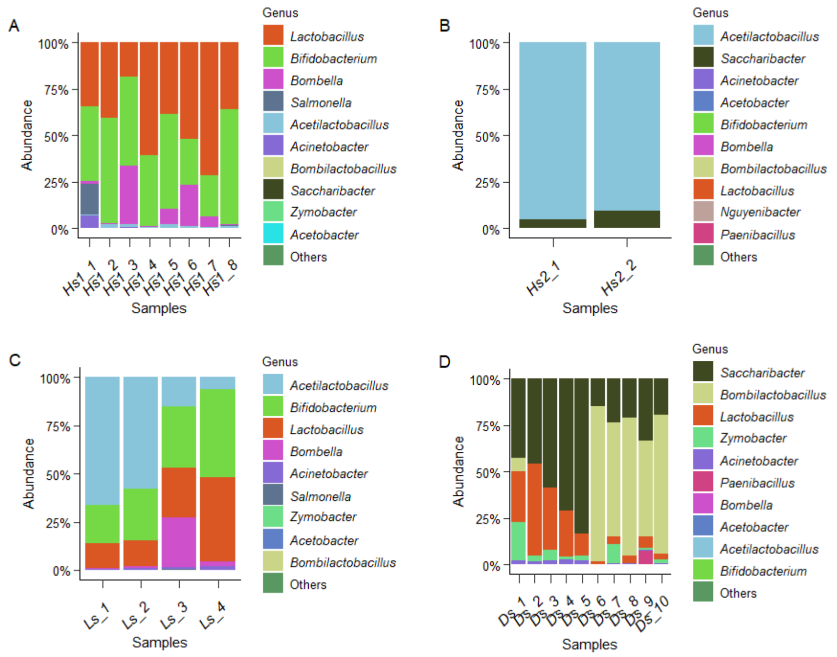

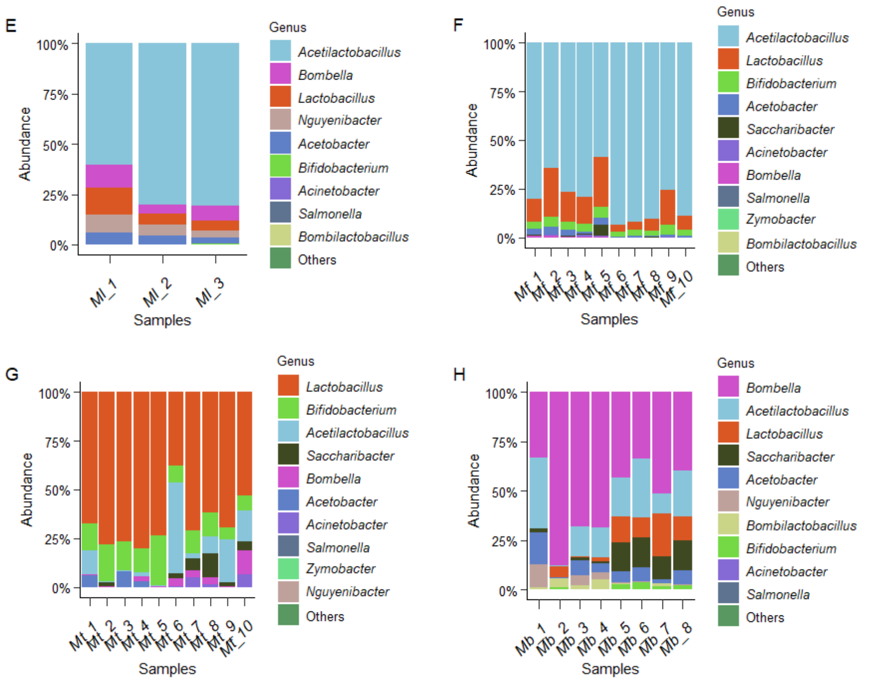

Figure S2: Gut bacterial genera associated with each of the eight stingless bee species in Kenya.

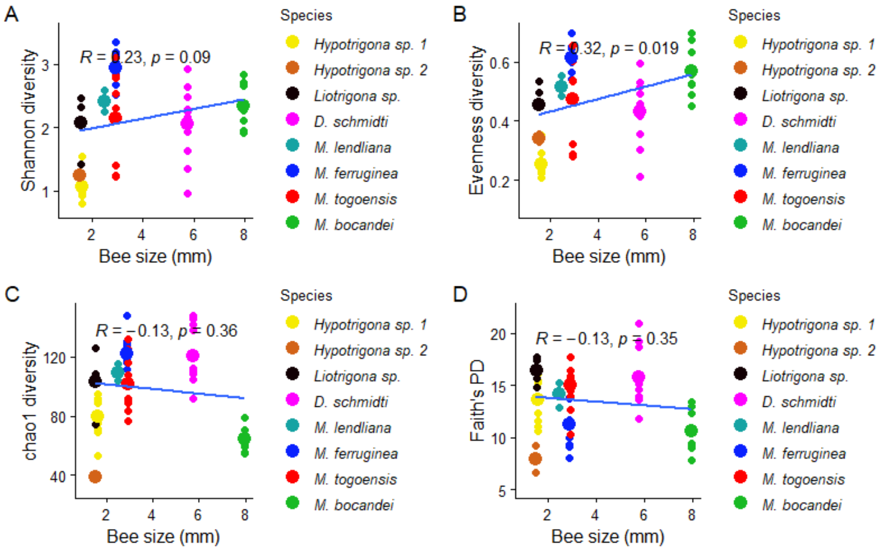

Figure S3: Bacterial alpha diversity did not correlate with the stingless bee size.

Table S1: 16S rRNA sequencing data analysis (Excel file).
